# Retraction: The Functional Influences of Common *ABCB1* Genetic Variants on the Inhibition of P-glycoprotein by *Antrodia cinnamomea* Extracts

**DOI:** 10.1371/journal.pone.0279301

**Published:** 2022-12-13

**Authors:** The PLOS ONE Editors

After this article [1, 2] was published, concerns were raised about the western blot images in Fig 1. Specifically, in Fig 1A:

- Lanes 3, 4, 6, 8 and 10 in the P-gp panel appear similar to each other, though not identical. Further assessment of this issue is limited by image quality.
- The first three lanes in the β-actin panel appear similar to lanes 4–6 and to lanes 7–9. Lane 10 in the β-actin panel in Fig 1A appears similar to lanes 1, 4 and 7.
- Editorial review identified the appearance of vertical discontinuities between lanes 3 and 4, between lanes 6 and 7, and between lanes 9 and 10 in the β-actin panel when the exposure of the image is adjusted.

In editorial follow up on these issues, the corresponding author stated that they did not intentionally rearrange the lanes relative to the original images, nor show the same data in multiple panels, and that recently repeated results demonstrated the same conclusions as the published data. The underlying data from the time of the original experiments for Fig 1A and 1B are no longer available.

In light of the above issues, which could not be resolved in the absence of the original underlying data, the *PLOS ONE* Editors retract this article.

MJS, YNT and CCH agreed with retraction and stand by the article’s findings. YYC either could not be reached or did not respond.
